# Paradoxical immune response in leishmaniasis: The role of toll‐like receptors in disease progression

**DOI:** 10.1111/pim.12910

**Published:** 2022-02-28

**Authors:** Ifeoluwa E. Bamigbola, Selman Ali

**Affiliations:** ^1^ Interdisciplinary Biomedical Research Centre School of Science and Technology Nottingham Trent University Nottingham UK

**Keywords:** immune response, *leishmania*, resistance, susceptibility, toll‐like receptor

## Abstract

Toll‐like receptors (TLRs), members of pattern recognition receptors, are expressed on many cells of the innate immune system, and their engagements with antigens regulate specific immune responses. TLRs signalling influences species‐specific immune responses during *Leishmania* infection; thus, TLRs play a decisive role towards elimination or exacerbation *of Leishmania* infection. To date, there is no single therapeutic or prophylactic approach that is fully effective against leishmaniasis. An in‐depth understanding of the mechanisms by which *Leishmania* species evade, or exploit host immune machinery could lead to the development of novel therapeutic approaches for the prevention and management of leishmaniasis. In this review, the role of TLRs in the induction of a paradoxical immune response in leishmaniasis was discussed. This review focuses on highlighting the novel interplay of TLR2‐ /TLR9‐driven resistance or susceptibility to 5 clinically important *Leishmania* species in human. The activation of TLR2/TLR9 can induce diverse anti‐*Leishmania* activities depending on the species of infecting *Leishmania parasite*. Infection with *L*. *infantum* and *L*. *mexicana* initiates TLR2/9 activation leading to host protective immune response, while infection with *L*. *major*, *L*. *donovani* and *L*. *amazonensis* trigger either a TLR2‐ /9‐related protective or non‐protective immune responses. These findings suggest that TLR2 and TLR9 are targets worth pursuing either for modulation or blockage to trigger host protective immune response towards leishmaniasis.

## 1 INTRODUCTION

Leishmaniasis is part of neglected tropical and subtropical diseases caused by flagellated protozoans belonging to the genus *Leishmania*.^1^ The genus consists of over 30 species distributed across different regions of the world. About 20 species are known to cause human diseases, 15 of which are zoonotic.^2^, ^3^ They are vector‐borne diseases, which are successfully transmitted by the bite of an infected sandfly into the host skin.^4^ The major species that causes human diseases are *L*. *tropica*, *L*. *major*, *L*. *aethiopica*, *L*. *mexicana*, *L*. *amazonensis*, *L*. *panamensis*, *L*. *guyanensis*, *L*. *peruviana*, *L*. *braziliensis*, *L*. *infantum* and *L*. *donovani*.^2^, ^3^ Leishmaniasis presents wide spectrum of clinical manifestations; however, three distinct clinical syndromes have been identified and they are: visceral leishmaniasis (VL), cutaneous leishmaniasis (CL) and mucosal leishmaniasis (ML).^5^

Over the past few decades, an exponential increase in the epidemiological burden of leishmaniasis has been reported with a strong link to poverty and poor health, thus presenting it as the fourth most prevalent tropical infections and ranked second by mortality rate.^6^ The disease is endemic to 98 countries affecting about 12 million people. A total of 350 million people are at the risk of infection with an approximate annual incidence of 2 million.^3^, ^6^, ^7^

The functional role of the human immune system is to orchestrate a quick and effective response to danger or infection induced by a pathogen, including bacteria, fungi, parasites and viruses.^8^ The innate immune system constitutes a non‐specific response to pathogens, while the adaptive immune cells provide late but highly specific response to antigens.^9^ Among the cells of the innate immune system involved in *Leishmania* infection are macrophages, neutrophils, dendritic cells, mast cells, basophils, eosinophils and natural killer cells, while the adaptive immune system is made up of T and B lymphocytes. Neutrophils, macrophages and dendritic cells are the most important functional cells of the innate immunity producing ranges of cytokines such as IFN‐γ, IL‐12 and TNF‐α.^10^, ^11^

Leishmaniasis progression depends on efficient proliferation of the parasites intracellularly in the mammalian host. This proliferation is determined by the type and potency of immune responses which can either interfere with or enhance the establishment of leishmaniasis. Thus, the *Leishmania–*host interactions present a complex paradoxical relationship. The innate immune system uses pattern recognition receptors (PRR) such as toll‐like receptors (TLRs), macrophage mannose receptors (MMR), NOD‐like receptors (NLR) expressed on antigen‐presenting cells (APC) for initial recognition of parasites pathogen‐associated molecular patterns (PAMP).^12^ Of these PRR, TLRs are first receptors to recognize *Leishmania*‐associated PAMPs.^12^ PRR signalling initiates several innate immune responses such as the activation of complement cascades, inducing phagocytosis, as well as the production of pro‐inflammatory cytokines.^12^

In response to host protective immune response, pathogens have developed numerous strategies to conquer the immune machinery.^13^ Evasion of innate immunity by *Leishmania* parasites is a critical step in their survival. The ability to avoid or suppress anti‐microbicidal factors produced by innate immune cells is a major evasion strategy employed by *Leishmania*. Further, *Leishmania* intracellular promastigotes adopt an adaptive lifestyle that helps them survive in host cells by remodelling the phagosomal compartment and interfering with signalling pathways that mediate parasitic clearance.^13^ Additionally, *Leishmania* parasites survive in host cells by interfering with toll‐like receptors signalling pathways which either disrupts immune homeostasis or renders immune cells inactive.^14^

Therefore, this review summarizes the paradoxical interaction that exists between host innate immune machinery and *Leishmania* parasites. Most importantly, this review explores and discusses the significance of TLR2 and TLR9 as a crucial factor in determining infection outcome across *Leishmania* species. A better understanding of the innate immunological response to *Leishmania* infection and its role in parasite survival is crucial to develop novel therapies.

## 2 PARADOXICAL IMMUNITY—LEISHMANIA PARASITE INTERACTIONS

### 2.1 Neutrophil–*leishmania* interaction

Neutrophils constitute the first line of immune cells to be deployed within the first few hours to the site of *Leishmania* infection.^15^ Their early recruitment is pivotal to early containment of infection.^15^ Neutrophils facilitate immune response through the modulation of several activities, including the engulfing of *Leishmania* promastigotes, the production of several arrays of antimicrobial factors such as neutrophil extracellular traps (NETs), lytic enzymes,^16^ reactive oxygen species (ROS)^17^ and differential cytokine production.^18^, ^19^ Orchestrated neutrophil immune responses to leishmania infection are modulated by TLRs. Studies have shown that TLRs mediate the early/appropriate recruitment of neutrophils to the site of infection, as well as neutrophils activation and their apoptosis.^20^, ^21^

It is worth noting that neutrophil involvement is not limited to the promastigote‐mediated phase of infection but extends well into the later phase of infection.^22^ Second‐wave deployment of neutrophils in *L*. *major*‐infected C57BL/6 (resistant) mice has been observed 7 days post‐infection.^23^ Further, Daboul^24^ has reported the presence of neutrophils in lesions of 56 patients with late‐stage cutaneous leishmaniasis caused by *L*. *major* in Damascus, Syria. The potency of neutrophil response against *Leishmania* may be an indicative of the phase of infection. While neutrophils are involved in the internalization of both amastigotes and promastigotes of *L*. *amazonensis*, the internalization of promastigotes is comparatively more efficient, resulting in TNF‐α‐mediated parasite clearance accompanied by the production of pro‐inflammatory cytokines such as IL‐12.^15^, ^22^.

Nevertheless, besides the protective role of neutrophils against *leishmania* infection, neutrophils can serve as a Trojan horse transiently spreading infective promastigotes or amastigotes to macrophages.^25^ This is achieved by the recruitment of neutrophils to site of infection without activating their lethal antimicrobial factors, uptake of apoptotic cells and hijacking the tendency of early death of neutrophils for recruitment of macrophages.^25^ For instance, *L*. *infantum* activated neutrophils migration as well as intracellular effector mechanisms, thereby inducing uptake of promastigotes. However, minimal release of neutrophil extracellular traps allows the survival of some intracellular promastigotes with active proliferative capacity.^26^ Similarly, *L*. *mexicana* amastigotes were rapidly internalized by neutrophils; nevertheless, parasitic uptake was relatively silent resulting in death of few parasites. This occurred because *L*. *mexicana* amastigote did not trigger ROS production but induces high expression of CD62L which inactivates neutrophilic immune response.^27^ This hypothesized the role of neutrophils as a Trojan horse which has been observed in several experimental models involving different species of *Leishmania parasite* (reviewed in Table 1).

**TABLE 1:** Neutrophil Trojan horse mechanisms during Leishmania Infection

**Interaction**	**Outcome**
**1.1 *Leishmania major***	
Parasite's promastigotes mimic apoptotic cells by expressing phosphatidylserine	This leads to intracellular survival of parasites via PMN inducing the production of TGF‐β while downregulating the production of TNF‐α^28^
Upregulating the release of leukotriene B4 and decreasing the production of lipoxin A4 by neutrophils	Modulating recruitment of anti‐inflammatory lipid mediators such as leukotriene B4 (LTB_4_) and lipoxin A4 LXA_4_)favouring parasite persistence^29^
**1.2 *Leishmania mexicana***	
Early recruitment of neutrophils to site of infection in infected C57BL/6 mice	Ingestion of parasites and formation of NETs; however *L*. *mexicana* exploits the early recruitment to block the induction of a protective immune response by impairing recruitment of monocytes and dendritic cells using neutrophils as a safe transient shelter. This contributes to the development of chronic lesions^30^
Amastigotes internalization with silenced parasitic uptake by neutrophils	Minimal killing of parasite resulting in persistence replication of amastigotes^27^
**1.3 *Leishmania amazonensis***	
Hydrolysis of NETs DNA framework by parasitic enzyme 3’NT/NU	Evasion of NETs favours progression of infection^31^
**1.4. *Leishmania donovani***	
Ingestion of promastigotes by lysosome‐independent compartment of neutrophils	Transiently transmitting parasites to macrophages^32^
Parasites LPG induces autophagy	Generation of ERK, phosphoinositide 3‐kinase and NADPH oxidase‐mediated ROS, increased engulfment of parasite by neutrophils, thus promoting transient transfer of parasite to macrophages^17^

### 2.2 Macrophage–*leishmania* interaction

Upon successful infiltration of neutrophils by *Leishmania* parasite, macrophages provide the next line of defence for the host, by inducing secretion of pro‐inflammatory cytokines (IL‐1, IL‐6, IL‐12 and TNF) and nitric oxides.^33^ Once they are recruited, free parasites and infected PMNs are phagocytosed; hence, macrophages become the decisive host cells for parasitic persistence and infection establishment as majorly of leishmania parasites differentiate into intracellular infective form (amastigotes) in macrophages.^34^

Further, ingestion of promastigotes by macrophages is a process mediated by several receptors including toll‐like receptors (TLR), complement receptors (CR), kinases and transcription factors.^33^, ^35^ Many of these mediators might negatively impact innate immunity signalling pathways, thus, resulting in deactivation of macrophages, favouring infection progression. For example, in the studies of Ghosh et al.^36^ and Guizani‐Tabbne et al.^37^ *L*. *donovani and L*. *major* evade host macrophages by suppressing nuclear factor‐kappa B (NF‐kB), an essential (transcription factor) in host defence which regulates the expression of several essential antimicrobial molecules. Similarly, *L*. *major* suppresses macrophages production of IL‐12 by inducing the expression of monarch‐1 molecule found on macrophages which negatively regulates NF‐kB.^38^ During infection of macrophages by *Leishmania*, the parasite can cause the blockade of active p65/p50 as well as inducing the p50/p50 repressor causing the effective blockade of IFN‐ɣ‐mediated NO production by macrophages.^39^, ^40^, ^41^

In a similar scenario, *Leishmania* parasite surface molecules are potent tools used by the parasite to counter macrophagic response. For instance, several studies have reported how *Leishmania* subvert macrophages microbicidal arsenal by using lipophosphoglycan (LPG), the most abundant virulent surface molecules produced by *Leishmania* to target phagosome membrane and maturation.^42^, ^43^ LPG disrupts macrophage cytoskeleton by mediating the accumulation of periphagosomal F‐actin.^44^ While by its ability to impair the recruitment of synaptotagmin V, an endosomal protein crucial to phagocytosis, LPG reduces phagocytic capacity of host membrane.^45^

Nevertheless, *Leishmania* host surface receptors are recognized by pathogen recognition receptors, especially toll‐like receptors to induce innate immune response. For example, toll‐like receptors on macrophages recognize LPG of *L*. *infantum* and *L*. *braziliensis*, thereby inducing the production of nitric oxide (NO).^46^ From the above evidences, it is safe to conclude that macrophage–*Leishmania* interaction also presents a paradoxical interaction. Hence, the ability of macrophages to elicit either protective or non‐protective host immune response to *Leishmania* infection depends on the signalling cascade expressed during the active stage of infection. Table 2 below gives a summary of experimental reports of some signalling cascade involved in *Leishmania*–macrophages interactions.

**TABLE 2:** Paradoxical interaction of macrophages and Leishmania

*Leishmania* species	Interaction with Macrophages	Outcome	References
*L. major*	*Leishmania* engage CR3 to block macrophages from/ producing IL−12	Failed T helper 1 immune response, thus disrupting parasites clearance	^47^
	Inhibition of IL−12 production by inducing the expression of monarch−1	Parasite survival and persistence causing infection progression	^38^
*L. mexicana*	Expression of parasite's PKC causes phosphorylation of downstream signalling protein	Increases internalization of parasites; however, PKC overexpression provides adaptation ability of the parasite to survive within the macrophage	^48^
*L. donovani*	LPG impairs recruitment of synaptotagmin V	Inhibition of phagolysosome biogenesis: proliferation of parasite	^42^
	Parasite induces activation of acid sphingomyelinase for rapid formation of ceramide in macrophages	Reduced parasite uptake Impair antigen presentation to T cells, thus inhibiting adaptive immunity to parasitic infection	^49^
	Parasitic induction of SHP−1 inhibits production of NO by macrophages	High intracellular parasitic load favouring parasites’ persistence	^50^
	Suppression of NF‐Kb	Evasion of macrophages for parasite survival	^37^
	Prevention of oxidative burst‐mediated apoptosis by induction of suppressor of cytokine signalling (SOCS) as well as overexpression of Thioredoxin and inhibition of IFN‐γ	Subversion of macrophage ROS‐apoptotic machinery Impairment of macrophage‐T cell crosstalk Continuous replication of parasite	^51^
*L. braziliensis*	Downregulation of amastin in parasite affects macrophages infectivity	Reduced amastigote persistence in macrophage: enhanced clearance of infection	^52^
*L. vianna* *L. braziliensis*	High production of nitric oxide (NO) by macrophage Low production of NO by macrophage	Significant level of parasitic phagocytosis: Mild infection establishment Escape of parasites from macrophages arsenal: High disease severity	^53^
*L. amazonensis*	Release and accumulation of nucleoside diphosphate kinase (Ndk) inhibits ATP mediated cytolysis of macrophages	Reduction of NO anti‐leishmanicidal action of macrophages Downregulation of extracellular ATP (eATP), inducing the production of nucleoside triphosphate (NTP) resulting in beneficial proliferation of *leishmania* promastigote and amastigote	^54^
*L. infantum*	Expression of ecto‐nucleotidases by parasites dampens macrophage activation	Favour infection establishment and progression	^55^

### 2.3 Dendritic cell–*leishmania* interaction

Activation and maturation of DC are triggered after recognition of danger signals called pathogen‐associated molecular patterns (PAMPs) by pattern recognition receptors (PRR) such as toll‐like receptor on DC (TLRs), C‐type lectin, simultaneously, concomitantly driving enhanced secretion of cytokines and the activation of naïve T cells for internalization of pathogens.^9^, ^56^ The initiation of protective immune response against *Leishmania* parasites depends on the transition of immature dendritic cells phenotypes to mature phenotypes. This transition is characterized by expression of CD40, CD80 and CD86 and production of pro‐inflammatory cytokine IL‐12.^57^

The mechanism of interaction of DCs with *Leishmania* parasites depends on species in question, parasite morphological status and host type.^4^, ^58^ For instance, dendritic cell receptor, DC‐specific ICAM‐3‐grabbing nonintegrin (DC‐SIGN) was actively involved in efficient phagocytosis of *L*. *mexicana* promastigotes by monocyte‐derived dendritic cells (moDCs) but silenced in interaction with parasite’s amastigotes, hence, reduced internalization of amastigotes ^58^. This does not rule out DC‐SIGN as a receptor for amastigotes, it only indicates the differential interaction of different *Leishmania* species with DC. In fact, *L*. *pifanoi* and *L*. *infantum* amastigotes bind more efficiently to DC‐SIGN than their promastigotes independently of LPG although it is an insignificant binding receptor for *L*. *major* promastigote.^59^ The bias in binding capacity of DC‐SIGN to different forms of different *Leishmania* species is suggestive of *Leishmania* tendency to evoke different modulating pathway when interacting with dendritic cells. This differential modulation of phagocytic cells by *Leishmania* species could either induce or hamper effective T helper 1 (TH1) immune response, thus explaining the diverse clinical pathologies of leishmaniasis.

*L*. *infantum* and some of its recombinant polypeptides induce the maturation of BMDCs with high expression of CD40, CD80 and CD86 co‐stimulatory molecules with concurrent IL‐12 production, thus inducing TH1‐type immune response.^19^ Similarly, interaction of *L*. *braziliensis* with dendritic cells upregulates their activation markers and led to the production of IL‐12 and TNF‐α ^60^. These novel observations are indication of the role of DCs to confer protective immune response against leishmaniasis. However, L*eishmania* parasites can inhibit TH1‐type immune response by impairing DC activation and maturation, consequently hindering the production of IL‐12, TNF‐α and γ, thereby presenting *Leishmania* parasites an escape mechanism dependently or independently of IL‐10 production.^60^, ^61^

Also, *L*. *amazonensis* impairs the activation and maturation of DC through the activation of adenosine A_2B_, increasing the production of cAMP and phosphorylation of extracellular signal‐regulated protein kinases 1/2 (ERK1/2).^61^ Further, *Leishmania* stalls DC maturation and avoid inflammasome activation especially the TLR/NF‐kB/NLRP3 axis by subverting the transcription factor landscape of DC. This favours infection establishment and immunopathology because the parasite causes a significant downregulation of gene expression related to pro‐inflammatory TLR signalling.^62^

## 3 TLR2 AND *LEISHMANIA* INFECTION

Similar to TLR1, 4, 5 and 6, TLR2 is expressed on the surface of cells such as neutrophils, macrophages, dendritic cells, B cells and T cells which actively recognizes microbial stimuli in contrast to TLRs 3, 7, 8, 9, 10, 11 and 13 which are expressed intracellularly in the endosomal compartment ^63^. TLR2 is inferably the most significant toll‐like receptor to *Leishmania* infection because it is most expressed in active stage of leishmaniasis as compared to other TLRs. TLR2 is centrally responsible for the recognition of lipophosphoglycan (LPG), the most expressed surface molecule of *Leishmania* parasites.^64^, ^65^

### 3.1 Role of TLR2 in *L. major* infection

In the study of Halliday et al.^66^ TLR2‐/‐ mice showed an increased susceptibility to promastigotes or amastigotes of *L*. *major* and *L*. *mexicana* infection, larger parasitic burden and pronounced large lesion size. TLR2 plays critical role in the control of cutaneous leishmaniasis while its absence augments TH2 responses resulting in exacerbated infection.^66^ Huang et al.^67^ co‐inject both genetically resistant C57BL/6 and susceptible BALB/c mice models with *L*. *major* and TLR2 agonist Pam3CSK4. A decreased parasitic burden in both mice models with no evidence of lesion development was observed. The observed reduced pathology of leishmanization in these mice models was due to efficient activation of DC and macrophages along with a significant production of pro‐inflammatory cytokines. Resistant to infection confers on conventional susceptible BALB/c mice illustrates the importance of TLR2 in effective clearance of *L*. *major* parasite clearance.

TLR2 and TLR4 are crucial receptors to initiation host defences against *L*. *major* infection; however, TLR2 is more expressed on the macrophages of patients with self‐healing lesion than those with non‐healing lesion when compared to TLR4 expression.^68^ Since TLR2 signalling is dependent on MyD88 adaptor protein, MyD88‐/‐ mice were found to be more susceptible to *L*. *major* infection marked with larger lesions when compared to WT mice.^69^ However, the mechanism of susceptibility is dependent on parasite strains, while MyD88‐/‐ C57BL/6 mice infected with *L*. *major* IR75 strains show an increased susceptibility to infection as a consequence of non‐protective TH2 response. MyD88‐/‐ C57BL/6 infected with *L*. *major* LV39 strains susceptible to infection is due to impaired Th1 response.^70^

**FIGURE 1:**
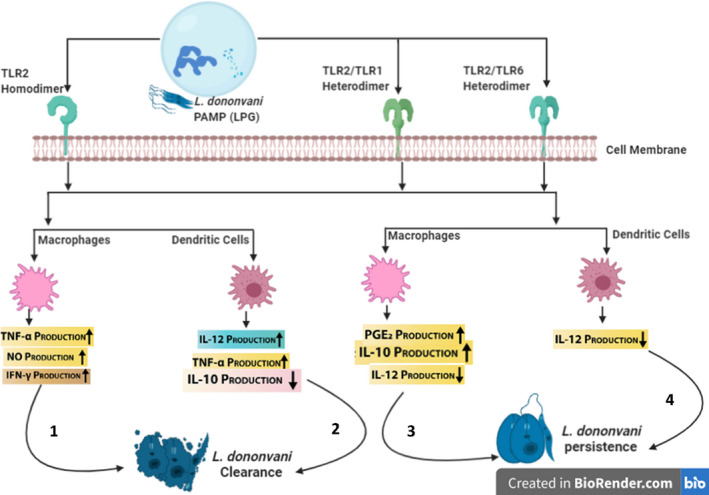
Role of TLR2 during L. major Infection (Figure created by BioRender.com) 1: Increased TLR2 expression during the course of L. major infection activates macrophages for the production of nitric oxide. 2–4: Maturation of dendritic cells induces the production of TH1 cytokines (IL‐12) while reducing TH2‐mediated response. 5–6: Overexpression of TLR2 promotes the establishment of non‐healing cutaneous leishmaniasis. Disease establishment is prompted by the downregulation of TLR4‐ and TLR9‐mediated protective immune responses

It is worthy to note that, despite the ability of TLR2 to form functional heterodimers with other TLRs, TLR2 plays the functional role against *L*. *major* independently of TLR1 and TLR6 (potential dimers) (Figure 1). Halliday et al.^66^ observed that TLR1 and TLR6 deficiency have no effect on disease kinetics of *L*. *major* infection.^66^

In total, evidences from the experimental studies described above are suggestive of the TLR2‐mediated protective immune response against *L*. *major* infection. Nevertheless, data from other studies hold great variability and the present mechanism by which TLR2 can promote disease establishment. For example, activated neutrophils contribution towards immunity against *L*. *major* infection has been demonstrated by stimulating differential production of pro‐inflammatory cytokines.^19^ However, Safaiyan et al.^71^ observed that neutrophils of patients suffering from non‐healing cutaneous leishmaniasis caused by *L*. *major* failed to induce the production of TNF‐α (Figure 1.), and this correlates with the high expression of TLR2 alongside TLR4 and TLR9.^71^ Hence, overexpression of TLR2 on neutrophils recruited to site of infection can exacerbate clinical manifestation with worsen prognosis.

Further, Srivastava et al.^72^ reported that *L*. *major* LPG activation of TLR2 enhances the survival of parasite in macrophages by reducing the expression of TLR4 and TLR9 while enhancing the expression of TLR1 and TLR11. However, this observation is subjective to the expression levels of LPG on *L*. *major* strains. In a bid to understand the underlying mechanism, the authors pre‐stimulate macrophages with peptidoglycan (PGN), a TLR2 ligand before infecting separately with either virulence or less virulent *L*. *major* strain and examine TLR9 expression as well as parasite survival in the macrophages. Data from the examination revealed that PGN enhanced survival of virulent strain parasites (with higher LPG content) in macrophages by inhibiting TLR9 expression. Therefore, it can be inferred that overexpression of LPG downregulates TLR9 expression by interacting with TLR2, thereby reducing anti‐leishmanial responses (Figure 1). This observation highlights the dual functionality TLR2‐mediated response to *L*. *major* infection and suggestive of the mechanism by which *Leishmania* LPG might be responsible for cutaneous leishmaniasis establishment in a TLR2‐dependent manner. Thus, it is possible that TLR2‐LPG interaction can induce the production of TH2 anti‐inflammatory cytokines such as IL‐10 as well TGF‐β, thereby reducing TH1 immune response.

Additionally, not only PGN‐TLR2 has been linked to *L*. *major* persistence in host cells, Pam3CSK4, a ligand of TLR1‐TLR2 heterodimer induces the production of more IL‐10 rather than IL‐12 and inhibits TLR9 expression in *L*. *major*‐infected macrophages. This results in TH2‐biased response which favours disease establishment. On the contrary, TLR2‐TLR6 heterodimer ligand, bisacycloxypropyl‐cysteine induces a TLR9‐dependent, IL‐12‐dependent as well as a regulatory T cell‐sensitive anti‐leishmanial protection ^72^. Hence, heterodimerization formed by TLR2 can modulate murine macrophages differently leading to different disease outcome.

### 3.2 Role of TLR2 in *L. mexicana* infection

Evidence has surfaced that TLR2‐/‐ mice not TLR1‐/‐ and TLR6‐/‐ mice are more vulnerable to *L*. *mexicana* infection, a vulnerability which is due to elevated production of anti‐inflammatory cytokines such as IL‐4, IL‐10 and IL‐13 by leukocytes in draining regional lymph nodes.^66^ Also, *L*. *mexicana* LPG activates natural killer T (NKT) cells by binding to TLR2 in initiation of defence against parasite, thereby activating DC of both resistant C57BL/6 and susceptible BALB/c mice strains for enhanced production of IL‐12 production.^73^ Cytokine analysis shows that DC of C57BL/6 mice produces more IL‐12 than BALB/c mice solely because of higher expression of TLR2 which correlates with NKT cells of C57BL/6 producing IFN‐γ when incubated with DC. Thus, TLR2 plays a crucial role in NKT cells induced protection against *Leishmania* during acute and innate phase of infection.^73^

Further, *L*. *mexicana* LPG induces the production of pro‐inflammatory cytokines including TNF‐α, IL‐12 and IL‐1 as well as activating ERK and p38 MAP kinase in human macrophages. However, silencing of TLR2 and TLR4 prior to macrophagic stimulation with parasite LPG resulted in reduced production of cytokine as well as suppressed phosphorylation of ERK and p38 MAP kinase ^74^. Therefore, TLR2 and TLR4 are the binding sites for *L*. *mexicana* LPG on macrophages to elicit effective immune response. Although silencing of TLR4 had a greater negative influence on cytokine production and kinases phosphorylation, silencing both receptors resulted in an almost complete inhibition of p38 MAP kinase phosphorylation, an indication of complimentary synergistic role between toll‐like receptors in innate immune response against *Leishmania* infection.

Additionally, patients suffering from diffuse cutaneous leishmaniasis (DCL) caused by *L*. *mexicana* are known to have a poorer prognosis compared with patients with localized cutaneous leishmaniasis (LCL) because they harbour low number of CD8 T lymphocytes in their lesions which is essential for infection clearance.^75^ Also, DCL patients CD8 T lymphocytes showed low cytotoxicity, antigen‐specific and IFN‐γ production when stimulated with autologous macrophages in vitro with *L*. *mexicana*. Interestingly, TLR2 agonist Pam3Cys or LPG restored the functional effector mechanisms of CD8 T lymphocytes while downregulating the expression of PD‐1 which is an indicator of lymphocytes exhaustion. This agrees with a recent study which reported that downregulation of TLR2 and JAK/STAT signalling is associated with NK cells dysfunction in diffuse cutaneous leishmaniasis ^76^. Hence, in addition to the role of TLR2 in innate immune response, it also regulates adaptive immune response towards leishmaniasis.^75^

### 3.3 Role of TLR2 in *L. infantum* infection

The extracellular expression of TLR2 for the production of adequate and efficient cytokines required for clearance *L*. *infantum* in canine monocyte‐derived macrophages is non‐negligible.^77^ In the study of Sacramento et al.,^20^ infection of BMDCs of WT mice with promastigotes of *L*. *infantum* showed a marked increase in the expression of TLR2 when compared to infected TLR2‐/‐ mice BMDCs. Also, TLR2 was significantly expressed in spleen and liver of infected WT mice 4 weeks post‐infection with high inflammatory infiltration and reduced parasite burden when compared to that TLR2‐/‐ mice. Combination of in vitro and in vivo data from this study demonstrates that *L*. *infantum* modulates TLR2 expression which participates in immune response against the infection. The authors reported that the absence of TLR2 affects DC maturation consequently affecting Th1 and Th17 protective immune response against *L*. *infantum* infection. TLR2 absence also impaired the recruitment and activation of neutrophils for the production of nitric oxide synthase, nitric oxide and TNF‐α, while IL‐10 production is upregulated. From these observations, it is evident that TLR2 signalling is crucial to confer enhanced protective adaptive immune response as well as anti‐parasitic function to neutrophils coordinated by DC production of CXCL1 during *L*. *infantum* infection.^20^

Similarly, TLR2 expression was found to be upregulated in blood samples of *L*. *infantum*‐infected dogs as compared to healthy ones. And a consequential reduction in the expression of TLR2 in the blood of the sample dogs after treatment with anti‐leishmanial agent.^78^ A clear indication that TLR2 was actively involved in innate immunity during high parasitic loads in the dogs. Moreover, it is believed that Ibizan hounds are more resistant to canine leishmaniasis caused by *L*. *infantum* infection and rarely show clinical manifestation of the disease, and this may be due to TLR2 expression. Martinez‐Orellana et al.^79^ observed that TLR2 agonist Pam3CSK4 successfully orchestrates higher production of TNF‐α in blood of Ibizan hounds stimulated alongside with *L*. *infantum* antigen when compared to those of seropositive dogs and healthy dogs.^79^

It has been elucidated that *L*. *infantum* SIR2RP1 (silent information regulator protein 1) protein modulation of B cells and induction of DC maturation to produce TNF‐α and IL‐12 is dependent on TLR2 signalling.^80^ However, emerging evidence implicates an association between TLR2 and TLR4 in coordinating innate immune response against visceral leishmaniasis. TLR2 and TLR4 are highly expressed on lymphocytes and monocytes of all patients with active VL in the study of Gatto et al.^81^ The expression of these receptors correlates with high production of TNF‐α, IL‐10 and NO before treatment with anti‐leishmanial drugs. Furthermore, TLR2‐4 expression persists after successful treatment with anti‐leishmanial drugs, this expression is accompanied by production of TNF‐α and NO. Observations suggestive of involvement of TLR2/4 in pathogenesis of VL and induction of protection against infection post‐treatment.^81^

### 3.4 Role of TLR2 in *L. donovani* infection

Prostaglandin E2 (PGE2) is an immunosuppressive compound produced within host macrophages aids *L*. *donovani* survival by inhibiting TH1 and upregulation of TH2 cytokines production by T cells.^82^ The generation of this immunosuppressive agent is subjective to the activation of enzymes cytosolic phospholipase A2 (cPLA2) and cyclooxygenase 2 (cox2). Stimulation of *L*. *donovani* infected macrophages with ligands of TLR1, 2, 3 and 4 prior infection led to a substantial increase in cPLA2 activation, thus indicating the significance of TLR in this immune signalling cascade. However, only the blockade of TLR2 signalling resulted in significant inhibition of cPLA2 activation, suggesting it is an indispensable binding site for *L*. *donovani* LPG to evade innate immune activities via the upregulation of PGE2.^82^

**FIGURE 2:**
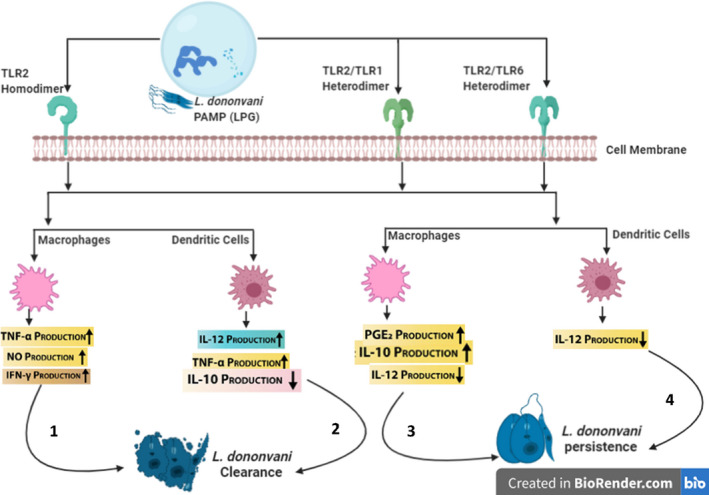
Role of TLR2 during *L*. *donovani* Infection (Figure created by BioRender.com). 1–2: TLR2 induces the production of anti‐leishmanial molecules by murine macrophages and dendritic cells for early clearance of *L*. *donovani* infection.^60^ 3–4: *L*. *donovani* counter protective immune mice response in BALB/C mice by macrophages and dendritic cells by the production TLR2‐dependent immunosuppressive molecule (PGE2). This causes a shift paradigm towards TH2 cytokine production^53^

Likewise, Chandra and Naik^83^ have observed that *L*. *donovani* significantly suppressed IL‐12 and upregulated IL‐10 production in TLR2‐stimulated macrophages and monocytes when compared to TLR4. This observation elucidates another strategy of how *L*. *donovani* evades innate immune response by enhancing ERK1/2 phosphorylation and suppression of P38 MAPK causing disruption ofTH1 and TH2 response homeostasis.^83^ No wonder, pre‐treatment of *L*. *donovani* infected macrophages with Arabinosylated lipoarabinomannan (Ara‐LAM), a TLR2‐dependent immunoprophylactic shifts the TH1/TH2 imbalance response towards protective TH1 via the upregulation of IL‐12 production and reduction in IL‐10 production.^84^ Ara‐LAM has also restored impaired splenic CD8^+^T cells proliferation in *L*. *donovani* infected BALB/c mice and improved IFN‐γ responsiveness to infection.^85^ Similarly, the study of Chowdhury et al.^86^ demonstrates that Ara‐LAM restored IFN‐γ responsiveness in infected *L*. *donovani* macrophages and potentiates elimination of parasites (Figure 2).

Similar to Chandra and Naik^83^ observation, data from Srivastav et al.^87^ agree that *L*. *donovani* infected BMDM failed to induce production of IL‐12 and TNF‐α despite carrying LPG, a ligand of TLR2 on its surface. However, the authors explained a completely different strategy through which *L*. *donovani* escape host’s immune phagocytic surveillance. Their study demonstrated that *L*. *donovani* reduced ubiquitination of TRAF6 (TNF receptor‐associated factor 6) while deubiquitinating a negative regulator enzyme of TLR signalling named A20. A20‐specific siRNA was found to restore the ubiquitination of TRAF6 alongside IL‐12 and TNF‐α production, concomitantly decreasing anti‐inflammatory cytokines (IL‐10 and TGF‐β) in *L*. *donovani* infected macrophages. Moreover, silencing of enzyme A20 in BALB/c mice model infected with *L*. *donovani* promastigotes resulted in increased NF‐kB activation. This led to restoration of pro‐inflammatory cytokines response, thus, efficient clearance of parasites. Aggregation of these observations suggests *L*. *donovani* can inhibit TLR2 signalling cascade by exploiting host A20.^87^

Evidence from other studies augments the fact that TLR other than TLR2 plays a protective role against *L*. *donovani* infection. For example, both TLR2 and TLR4 expressions were enhanced in livers of C57BL/6 *L*. *donovani* infected mice; however, they play contrasting roles.^88^ Murray et al.^88^ observed that TLR4‐/‐ infected mice showed reduced IFN‐γ, TNF and iNOS mRNA expression, thus presenting slow unresolving infection. Unlike infected TLR2‐/‐ mice presenting high parasitic clearance in liver because of enhanced pro‐inflammatory cytokine as well as reduced IL‐10 production.^88^ Similarly, despite the ability of TLR2 to activate phagocytic activation of macrophages due to *L*. *donovani* infection, silencing either TLR2 or TLR3 impairs the secretion of NO and TNF‐α post‐infection of IFN‐γ‐primed macrophages with *L*. *donovani* promastigotes.^89^

### 3.5 Role of TLR2 in *L. amazonensis* infection

The role of TLR2 in infection outcome by *L*. *amazonensis* has not been fully understood, as the existing literature describes wide range of effects of TLR2 in *L*. *amazonensis* infection.^90^, ^91^

Guerra et al.^90^ have observed that *L*. *amazonensis* infected TLR2‐/‐ mice show a low parasite burden and present greater resistance to infection when compared to C57BL/6 WT mice. The study shows that infected C57BL/6 WT mice orchestrate significant recruitment of inflammatory cells as compared to TLR2‐/‐ mice. However, TLR2‐/‐ mice present no free amastigotes and a reduced number of parasitized macrophages along with neutropenia during the infection period as opposed to what was observed in C57BL/6 mice. The observation shows that the absence of TLR2 signalling can cause alterations in immune cell profile and thus increases resistance of mice models to *L*. *amazonensis* infection.^90^

Contrastingly, *in vitro* infectivity index of *L*. *amazonensis* was much higher in BMDM of TLR2‐/‐ mice when compared to BMDM of C57BL/6 WT mice.^91^ In fact, TLR2‐ and TLR4‐mediated *L*. *amazonensis* recognition confers infectivity resistance on macrophages by upregulating Nos2 mRNA expression for nitric oxide production to kill parasites. Therefore, deficiency of these TLRs induces the production of polyamines which favours parasite replication.^91^ This observation represents the dichotomous nature of TLR2 in determination of the outcome of *Leishmania* infection.

It can be argued that the contrasting reports from the studies above are due to differences in experimental set‐up of these studies. Moreover, the study of Guerra et al.^90^ involved several inflammatory cells such as macrophages, neutrophils and eosinophils which could be synergistically working to wade of infection via TLR2 signalling, whereas the study of Muxel et al.^91^ focussed on TLR2 signalling in macrophage‐mediated immune response against *L*. *amazonensis* infection.

## 4 TLR9 AND *LEISHMANIA* INFECTION

TLR9 recognizes CpG motifs of bacterial and virus genomes^92^; there is an evidence of crosstalk between *Leishmania* CpG DNA and TLR9 which plays role in initiating protective anti‐parasite responses.^93^, ^94^ In subsequent subsection, the crosstalk between TLR9 and different *Leishmania* species will be highlighted.

### 4.1 Role of TLR9 in *L. major* infection

**FIGURE 3:**
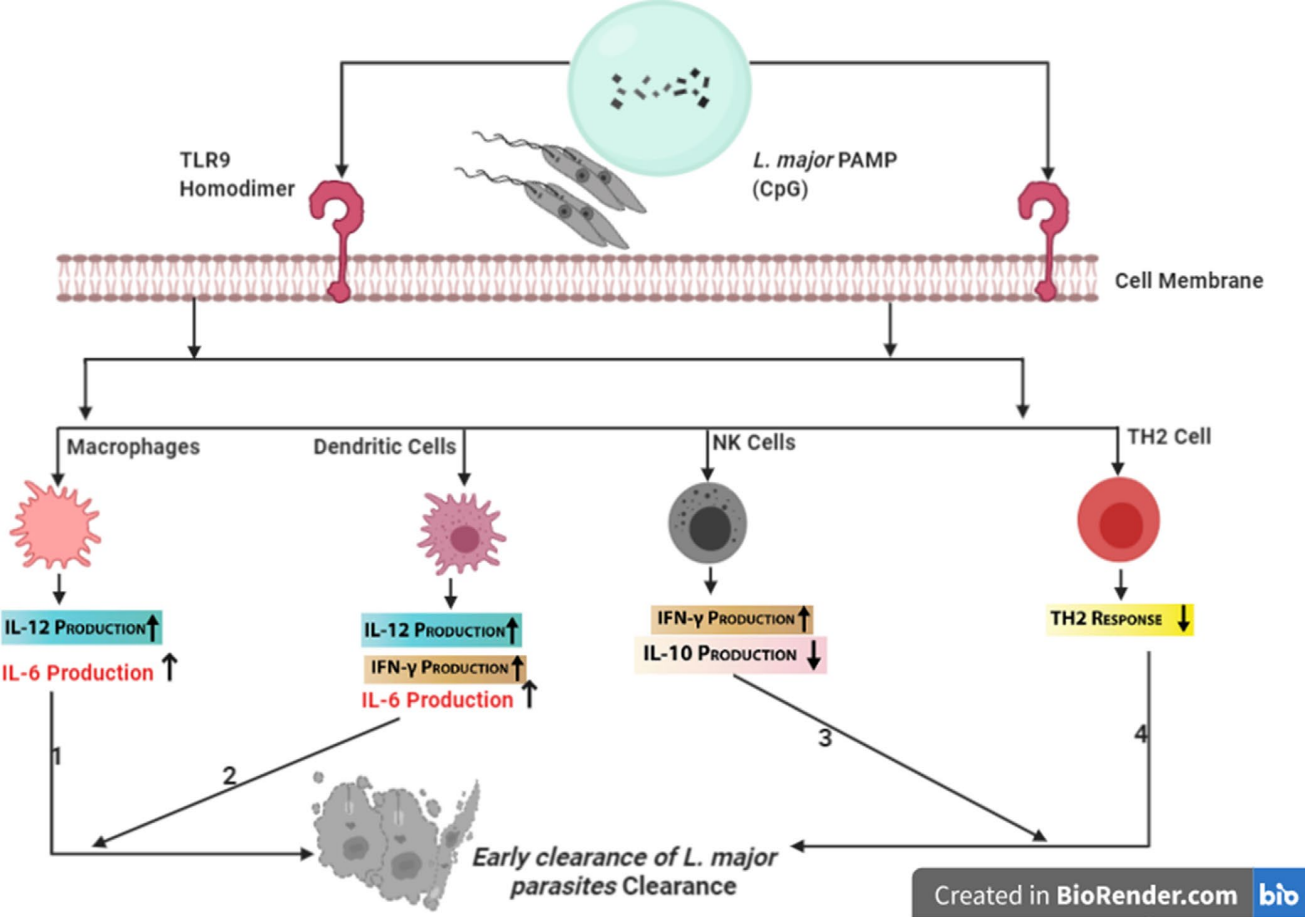
Role of TLR9 in L. major Infection (Figure created by BioRender.com). 1–4: TLR9‐mediated immune responses enhance the early clearance of L. major parasites in host cells by downregulation of TH2 immune response, thus increasing production of pro‐inflammatory cytokines by innate immune cells

To investigate the role of TLR9 in innate immune response against *L*. *major* infection, Liese et al.^94^ have infected TLR9‐/‐ mice and C57BL/6 WT with parasite promastigotes. TLR9‐/‐ infected mice exhibited progressive lesions and higher parasites burden during the acute phase of infection as compared to infected C57BL/6 WT mice. Further, data from analysis of cytokine mRNA expression showed that IFN‐γ was rapidly and effectively upregulated in draining lymph nodes (LN) of WT mice. The expression of this cytokine was significantly reduced in draining LN of TLR9‐/‐ mice. Since natural killer (NK) cells are important source of IFN‐γ production; thus, these data support the hypothesis that early response of NK cell to *L*. *major* infection is dependent on TLR9 signalling (Figure 3).^94^ While IL‐12 production is indispensable during NK cell‐mediated immune response, the authors studied the production of IL‐12 by in vitro stimulating BMDC from the two mice models. Stimulated BMDC of TLR9‐/‐ failed to orchestrate the production of IL‐12. This further substantiates the importance of TLR9 in effective clearance of *L*. *major* infection.

In fact, comparing the susceptibility of TLR2‐/‐, TLR4‐/‐, TLR9‐/‐ and C57BL/6 WT to *L*. *major* infection, TLR9‐/‐ mice are most susceptible to *this* infection. This is due to aberrant TH2 response resulting in low production of IL‐12 while increasing the production of IL‐10 at draining LN as opposed to TH1 immune response in WT mice.^95^ Further, in vitro stimulation of BMDCs of TLR9‐/‐ mice with *L*. *major* did not upregulate CD40 and CD80 resulting in failed generation IL‐12, IL‐6 and IFN‐β. This report agrees with that of Liese et al.^94^; however, they observed TLR9 deficiency did not prevent ultimate resolution of infection. This suggests that, although TLR9 signalling contributes to the maturation of dendritic cells, activation of NK cells and production of pro‐inflammatory cytokines for early parasitic clearance, its role is dispensable for a protective T cell response.

### 4.2 Role of TLR9 in *L. donovani* infection

*L*. *infantum* infection modulates TLR9 expression on the surface of dendritic cells which suggest that this receptor may be involved in the recognition of the parasite and thus initiate protective immune response against the parasites.^96^ Therefore, an impaired or failed expression of TLR9 could favour parasites’ persistence in host cells. Sacramento et al.^96^ further highlighted the significance of TLR9 for effective control of *L*. *infantum* infection in mice models. TLR9‐/‐ infected mice show increased susceptibility to infection marked with enhanced parasitic burden in livers and spleen when compared to WT mice, thus validating the role of the receptor in protective response against *L*. *infantum* infection. Further, neutrophil recruitment to inflammatory foci during *L*. *infantum* infection is dependent on TLR9 signalling. TLR9‐/‐ infected mice show a decrease in neutrophil recruitment to inflammatory foci due to defect in the production of chemotactic receptors. This impairment of neutrophil recruitment during acute stage of infection affects TH1‐ and TH17‐mediated immune response ^96^.

Similarly, DC activation leading to the production of IL‐12 in response to *L*. *infantum* infection requires TLR9 signalling.^94^ The authors also reported that NK cell activation for the production of IFN‐γ is dependent on TLR9 signalling. Their hypothesis was confirmed by a comparative study on infected mice models. Leishmanization of WT mice was rapidly followed by NK cell activation in the spleen with induction of IFN‐γ production. In contrast, *L*. *infantum*‐induced NK cell activation was abolished in TLR9‐/‐ mice.^94^

### 4.3 Role of TLR9 in *L. donovani* infection

Till date, very little is known about the role of TLR9 during *L*. *donovani* infection. However, it significantly recognizes *L*. *donovani* CpG DNA,^93^ with possibility of initiating TH1‐mediated immune response. Further, miltefosine treatment has shown to reduced intracellular parasite load *in L*. donovani infected THP‐1 cells, triggering a strong inflammatory cytokine response involving IFN‐γ, IL‐12 and TNF‐α. This pool of cytokines produced to wade off VL is accompanied by significant expression of TLR4 and TLR9.^97^ These changes in the TLR expression and cytokine expression were also noticed in peripheral blood mononuclear cells of VL patients treated with miltefosine. These observations suggest the possible dependence of miltefosine anti‐leishmanial activity on either TLR4 or TLR9 signalling. These agree with the report of Babiker et al.^98^ who found the expression of TLR4 and TLR9 alongside cytokines expression in blood samples of active patients with VL in Sudan.

### 4.4 Role of TLR9 in *L. amazonensis* infection

There is no significant difference in the infectivity of *L*. *amazonensis* amastigotes and NO production in macrophages in vitro of TLR9‐/‐ mice and that of resistant C57BL/6 wild mice. Further, the ability of neutrophils from both mice models to produce NETs in response to *L*. *amazonensis* infection is similar. Although *L*. *amazonensis* failed to activate dendritic cells in both models, TLR9‐/‐ mice are more susceptible to *L*. *amazonensis* in vivo infection than WT. Susceptibility is marked with larger lesion and increased parasite burden during chronic stage infection due to decreased IFN‐γ production in infected tissue as well as increased IgG production.^99^ Taken together, the result of this study suggests that TLR9 contributes to C57BL/6 mice resistance to *L*. *amazonensis* infection. However, TLR9 signalling can promote progression of cutaneous lesions as well as promote intracellular survival of *L*. *amazonensis* by inducing the expression of CD200, a ligand known for suppressing pro‐inflammatory cytokines production by macrophages.^100^

## 5 CONCLUSION

The significance of toll‐like receptors in the complex paradoxical *Leishmania*‐innate host cell interaction was reviewed. It appears that activation of toll‐like receptors can be involved in either host protective or non‐protective immune responses depending on *Leishmania* species, TLR receptor heterodimerization and the elicited differential immune cascades. Activation of TLR2/TLR9 induces a protective immune response to *L*. *infantum* and *L*. *mexicana* infection while the activation of TLR2/TLR9 can either promote a host protective or non‐protective immune response to *L*. *major*, *L*. *donovani* and *L*. *amazonensis* infection. Despite this complexity, a great deal of data highlights the importance of balance between TH1/TH2 immune response during *Leishmania* infection to confer host resistance. Though, the trick of trade by which *Leishmania* influences the plasticity of toll‐like receptors dependent immune responses is yet to be well elucidated. Targeting TLR2 and TLR9 signalling pathways may be important in modulating responses to *Leishmania* parasite infection.
